# Thermal Decomposition of 2-Cyclopentenone

**DOI:** 10.1021/acs.jpca.4c05532

**Published:** 2024-10-15

**Authors:** Kathryn Narkin, Heather R. Legg, Glenna J. Brown, Khaled El-Shazly, Thaddeus D. Martin, Mia Jarrell, Laura R. McCunn, Zhijian Chen, Carol A. Parish

**Affiliations:** † Department of Chemistry, Marshall University, 1 John Marshall Dr., Huntington, West Virginia 25755, United States; ‡ Department of Chemistry, University of Richmond, Gottwald Center for the Sciences, Richmond, Virginia 23173, United States

## Abstract

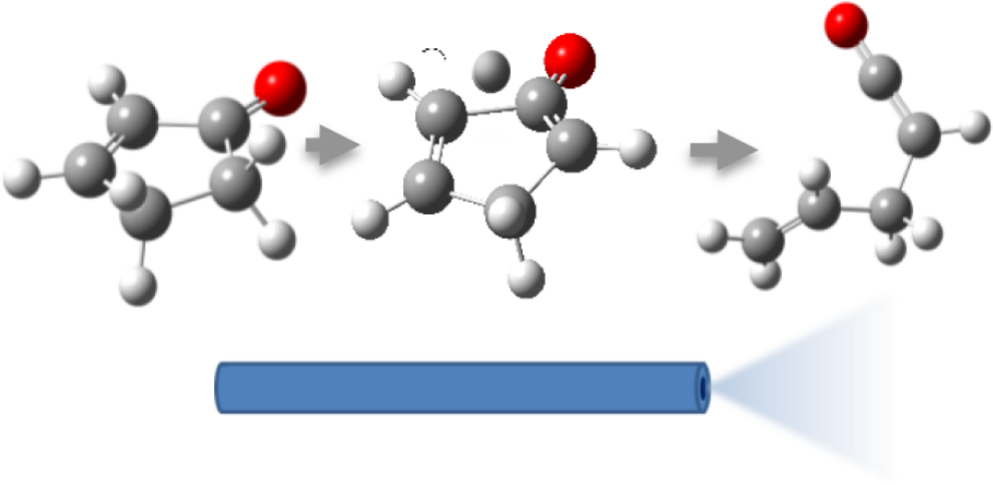

The thermal decomposition of 2-cyclopentenone, a cyclic oxygenated hydrocarbon that occurs in the pyrolysis of biomass, has been studied in a combined experimental and theoretical approach. Gas-phase pyrolysis was performed at temperatures ranging from 1000 to 1400 K in a pulsed, microtubular reactor. Products were identified by FTIR spectroscopy following their isolation in a low-temperature argon matrix. The following products were identified: carbon monoxide, ketene, propenylketene, vinylacetylene, ethylene, propene, acrolein, acetylene, propyne, and propargyl radical. Computational results identify three different decomposition channels involving a H atom migration, and producing prop-2-enylketene (Pathway 1), prop-1-enylketene (Pathway 2), and a second conformation of prop-2-enylketene (Pathway 3). A fourth decomposition pathway involves simultaneous rupture of two C–C bonds forming a high energy cyclopropenone intermediate that further reacts to form ethylene, acetylene, and carbon monoxide. Finally, a fifth pathway to the formation of acrolein and acetylene was identified that proceeds via a multistep mechanism, and an interconversion from 2-cyclopentenone to 3-cyclopentenone was identified computationally, but not observed experimentally.

## Introduction

The pyrolysis of biomass produces solid biochar, liquid bio-oil, and many gases, including CO and H_2_.^1−4^ While all three phases have valuable applications, the bio-oil holds great potential for refinement into usable biofuels and chemical feedstocks. Bio-oil can contain hundreds of different compounds, including water, acids, alcohols, aldehydes, ketones, esters, phenols, carbohydrates, furans, aliphatic and aromatic hydrocarbons, and nitrogen-containing compounds.^5−7^ Cyclic, oxygenated hydrocarbons are common components of bio-oil and also occur as intermediates in the pyrolysis and combustion of biomass.^8−12^ Thus, it is critical to understand how cyclic oxygenated hydrocarbons decompose at high temperatures in order to understand the chemistry, efficiency, and environmental impact of biofuels derived from biomass.

**Figure 1:**
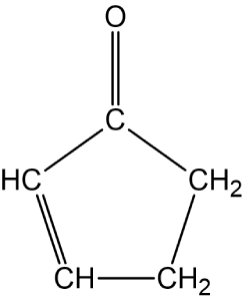
2-Cyclopentenone.

Cyclopentenones are cyclic ketones with a double bond in the five membered-ring, often detected in the pyrolysates of many forms of biomass such as wood, coffee grounds, algae, and peat deposits.^10,13−18^ GC/MS analysis has detected the isomer 2-cyclopentenone (Figure 1) in the biocrude oil produced from pyrolysis (673–873 K) of coffee grounds.^14^ It has also been detected in the pyrolysates of safflower residues,^19^ cactus spines,^20^ poplar sawdust and rice husk,^21^ pine wood chips,^17^ water hyacinth,^22^ sugar cane vinasse,^23^ and greenhouse vegetable waste mixed with coal.^24^ It is a component of wood smoke,^25^ liquid smoke from pine and cacao sawdust,^26^ the biocrude from hydrothermal liquefaction of food/vegetable solid waste,^27^ soybean stalk,^28^ and switchgrass,^29^ agricultural waste straw,^30^ and algal biomass.^15^ It is also seen in the hydrothermal liquefaction of l-alanine, indicating a potential role in protein decomposition.^31^ On a molecular level, it is known that 2-cyclopentenone can be formed from the reaction of O(^3^P) with cyclopentadiene. Quantum Monte Carlo and DFT calculations suggest that the O(^3^P) addition reaction proceeds by the formation of a triplet diradical, followed by an intersystem crossing and then traversing a transition state to 2-cyclopentenone.^32^ Lastly, 2-cyclopentenone has been shown to be an important product of cyclopentanone oxidation and decomposition.^33−36^

The purpose of this study is to understand the thermal decomposition of gas-phase 2-cyclopentenone as there are no published studies on the subject. An early study of gas-phase 2-cyclopentenone photolysis (238–265 nm) from 1967 detected carbon monoxide but no other products.^37^ Here, gas-phase pyrolysis in a small tubular reactor is coupled with matrix-isolation FTIR spectroscopy to identify the pyrolysis products at temperatures up to 1400 K. Calculations employing density functional theory reveal how the major products can be formed in unimolecular decomposition reactions. The combined experimental and theoretical approach provides important information for predicting the composition of biofuels manufactured from biomass pyrolysis. As the experiments here probe the reactions occurring early in the pyrolysis mechanism, they also provide important first clues to the development of an overall mechanism for biomass pyrolysis that includes 2-cyclopentenone as an intermediate.

## Methods

Thermal decomposition of 2-cyclopentenone (purity ≥98%; Alfa Aesar) was accomplished via a pulsed hyperthermal tubular reactor that has been described elsewhere in the literature.^38^ A mixture of 0.3% 2-cyclopentenone in approximately 1000 Torr ultrahigh purity argon was prepared using standard manometric techniques. The mixture entered a resistively heated silicon carbide (SiC) tube with a length of 38 mm and inner diameter of 1 mm through a pulsed valve (General Valve Series 9). The pyrolysis temperature (1000–1400 K) was controlled by a Series 16A temperature controller (Love Controls) and measured by a type C thermocouple mounted on the outside of the SiC tube. The effective temperature of the gas traveling through the tube will vary as a function of the distance traveled through the tube and the radius from the tube’s centerline. With argon as the carrier gas, the maximum gas temperature reached inside the tube could be estimated to be within 50 K of the wall temperature.^39,40^ Wall temperatures are reported for all experiments herein. The pyrolysis products exiting the tube were frozen in an argon matrix on a cesium iodide window mounted in a cryostat (Janis Research) with a base pressure of 1.0 × 10^–6^ Torr. The CsI window was maintained at a temperature of 15 K during deposition by a closed-cycle helium refrigerator (Sumitomo Heavy Industries Ltd.) and regulated by a Lake Shore 331 Temperature Controller. Prior to FTIR analysis, the window was cooled to 4 K. FTIR spectra were collected for 128 scans at 0.5 cm^–1^ resolution with a Bruker Vertex 70 spectrometer that was purged with dry air.

*Ab initio* calculations were used to identify possible unimolecular thermal decomposition reactions of 2-cyclopentenone and to support the assignment of vibrational spectra, using the Gaussian 16 software.^41^ Optimization and frequency calculations were performed on 2-cyclopentenone, reaction intermediates, transition states, and products at the B3LYP^42−45^ and 6-311++G** levels of theory.^46^ Hybrid functionals such as B3LYP have been shown to perform well when calculating geometries and barrier heights, especially when paired with large Pople style basis sets such as 6-311++G**.^47^ Vibrational frequency analysis was used to confirm that all transition states were first order saddle points connecting the expected structural minima. For all calculations a restricted wave function was utilized, except in the case of the diradical intermediate found in pathway 5 where an unrestricted approach was taken. Intrinsic reaction coordinate (IRC)^48,49^ calculations were also used to confirm all transition states and decomposition pathways.

## Results and Discussion

**Figure 2:**
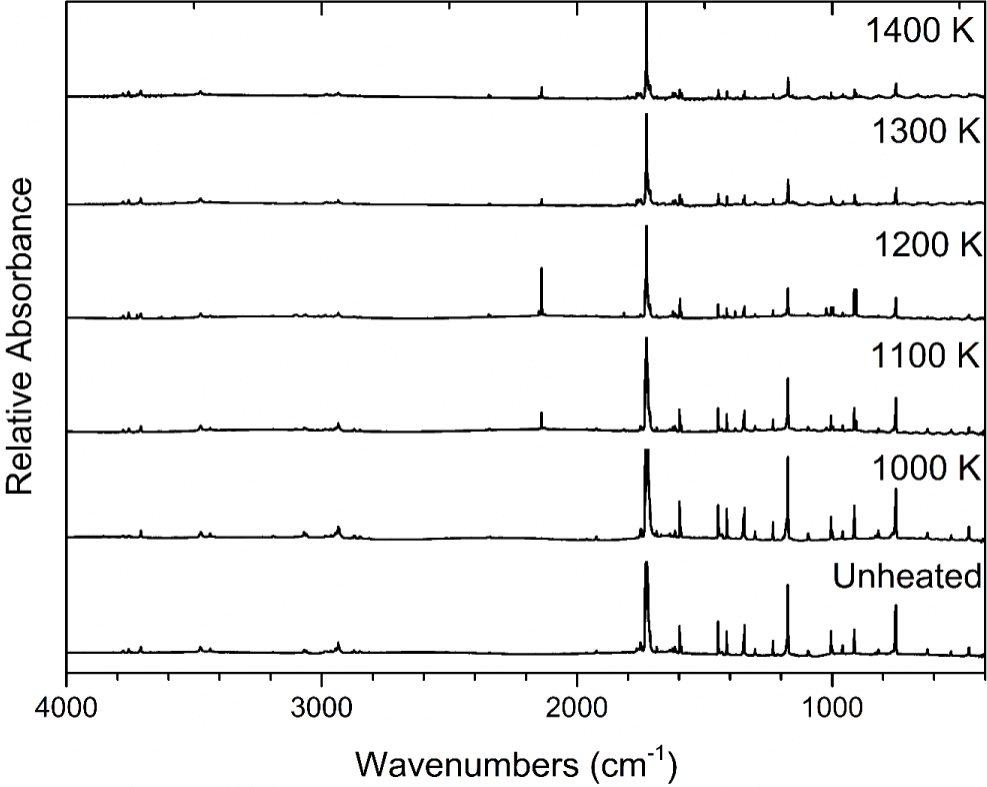
Temperature study of the pyrolysis of 2-cyclopentenone. All traces display matrix-isolation FTIR spectra of 0.3% mixtures of 2-cyclopentenone in argon, collected following pyrolysis at the indicated temperatures.

Pyrolysis was performed at several temperatures over the 1000–1400 K range. (Figure 2) Pyrolysis at 1300 K produced comparatively larger peaks and displayed the full range of products observed over all pyrolysis temperatures used in this study and thus, the discussion will focus on the spectrum acquired at that temperature. The assignment of products in the matrix-isolation FTIR spectrum following pyrolysis at 1300 K of 2-cyclopentenone was accomplished by comparing the experimental vibrational frequencies and their relative intensities to the values reported in literature for matrix-isolated species under similar conditions. The spectrum recorded following pyrolysis was also compared to the spectrum obtained of matrix-isolated 2-cyclopentenone, without pyrolysis, as well as a spectrum of pure argon deposited after heating to 1300 K in order to verify that the products were indeed the result of pyrolysis reactions of 2-cyclopentenone. It was found that pyrolysis of 2-cyclopentenone produces carbon monoxide, ketene, acetylene, ethylene, acrolein, prop-2-enylketene and/or prop-1-enylketene, vinylacetylene, propene, propyne, and propargyl radical.

**Figure 3:**
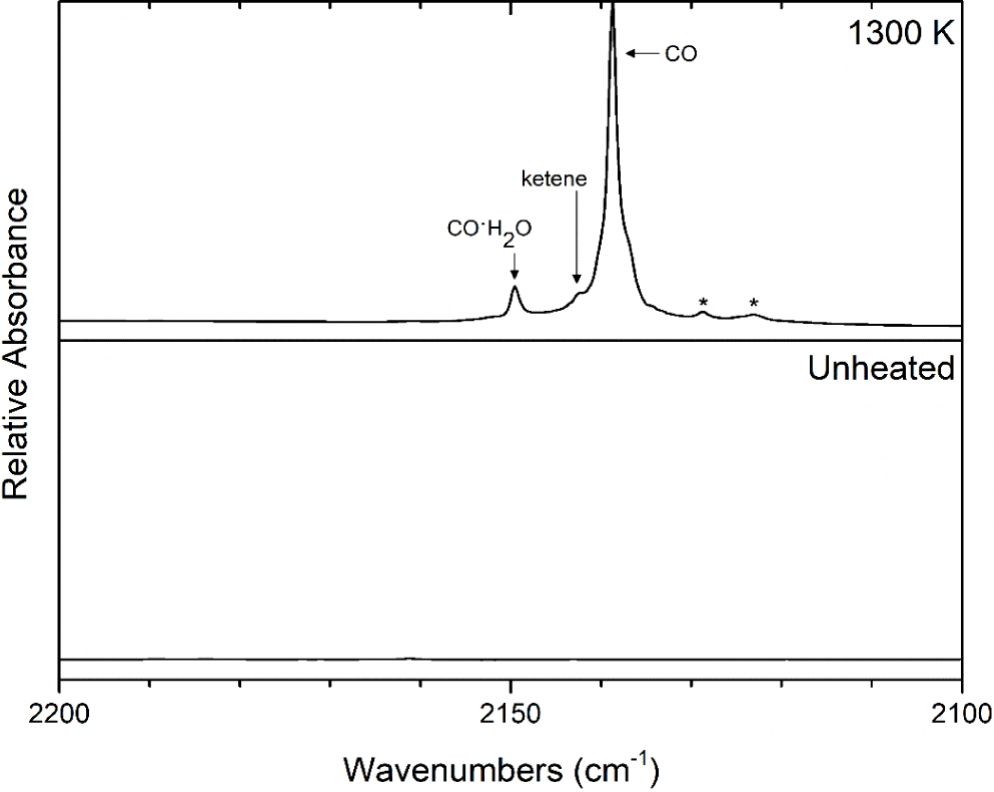
Matrix-isolation FTIR spectrum collected following 1300 K pyrolysis of 0.3% 2-cyclopentenone in argon (upper trace) compared to that of unheated 0.3% 2-cyclopentenone in argon. The asterisks mark substituted ketenes, purported to be propenylketenes, as described in the text.

**Figure 4:**
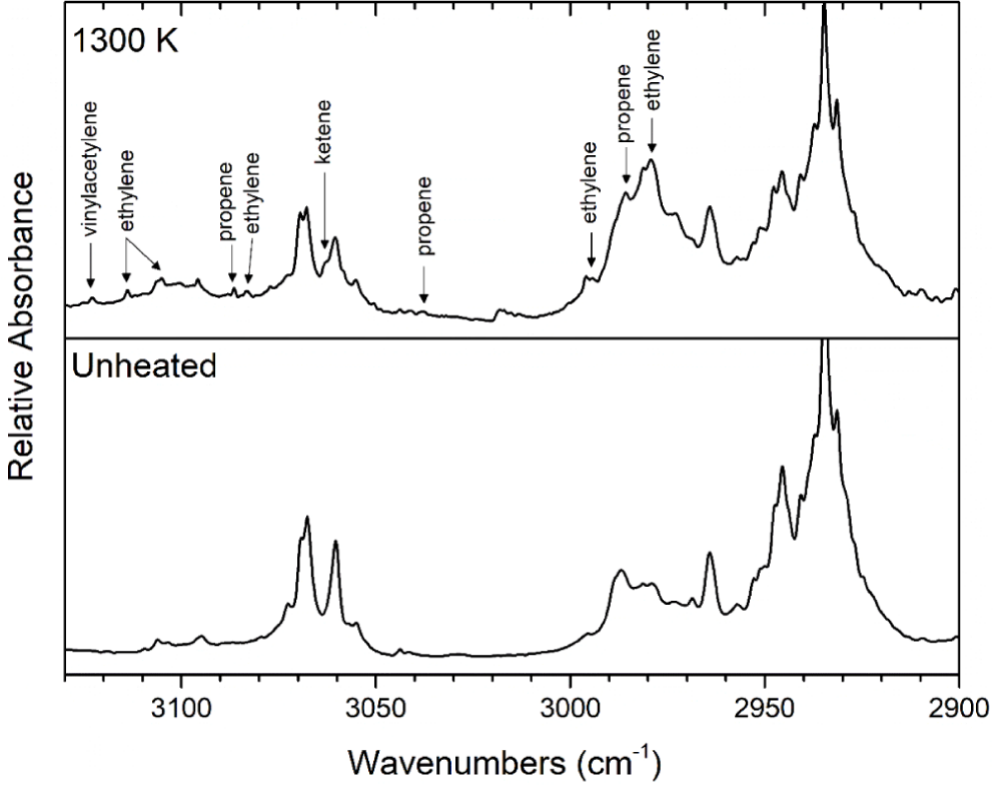
Matrix-isolation FTIR spectrum collected following 1300 K pyrolysis of 0.3% 2-cyclopentenone in argon (upper trace) compared to that of unheated 0.3% 2-cyclopentenone in argon.

**Figure 5:**
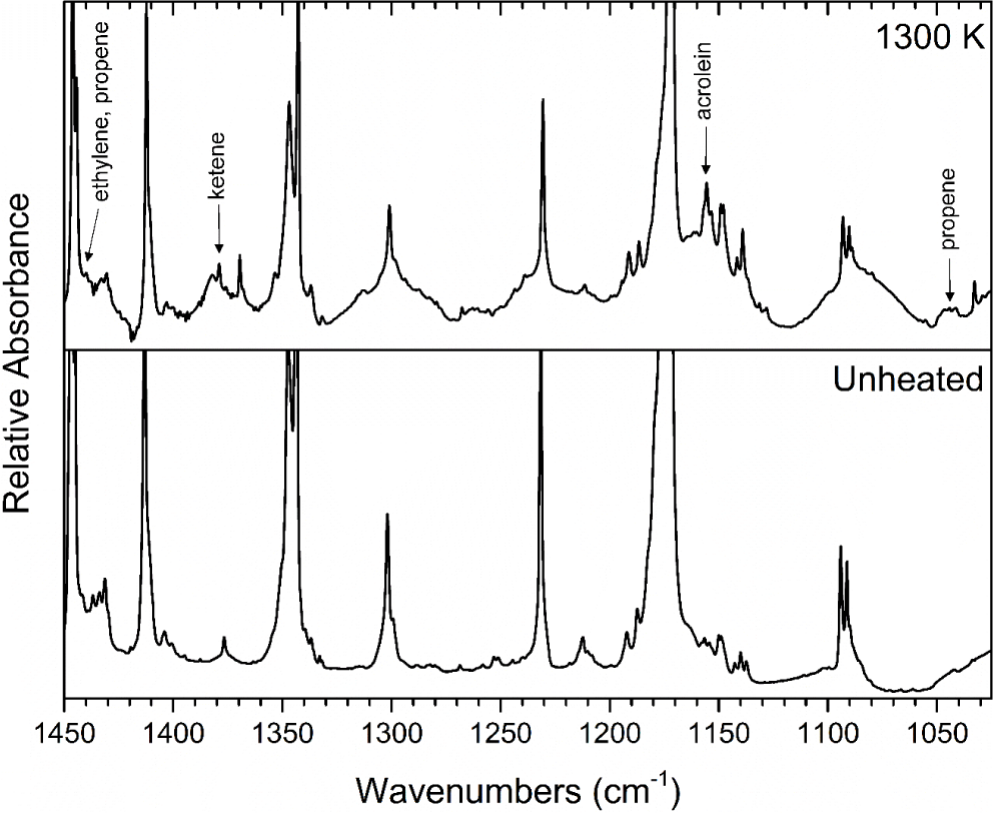
Matrix-isolation FTIR spectrum collected following 1300 K pyrolysis of 0.3% 2-cyclopentenone in argon (upper trace) compared to that of unheated 0.3% 2-cyclopentenone in argon.

Carbon monoxide was detected at 2139 cm^–1^, which matches the literature value.^50^ (Figure 3) The band found at 2149 cm^–1^ was determined to be a carbon monoxide-water complex (CO·H_2_O) and matches the value found in the literature.^50^ The complex is attributed to the clustering of CO with background water present in the cryostat. The signature *v*_2_ band of ketene was observed at 2142 cm^–1^. Based on literature intensities,^51^ the next most intense band of ketene would be expected at 3063 cm^–1^, but this band appears as a shoulder on the band of unreacted 2-cyclopentenone in the spectrum (Figure 4), however, the ketene band at 1380 cm^–1^, can be seen in Figure 5.

Bands at 2129 and 2123 cm^–1^ are marked with asterisks in Figure 3 and are of particular interest as they could not be assigned to any known compounds containing carbon, hydrogen and/or oxygen. The bands were hypothesized to correspond to substituted ketene(s) based on their proximity to the signature antisymmetric C=C=O stretch (*v*_2_) of ketene.^52,53^ Propenylketenes were considered as possible assignments because they are isomers of 2-cyclopentenone.

**Figure 6:**
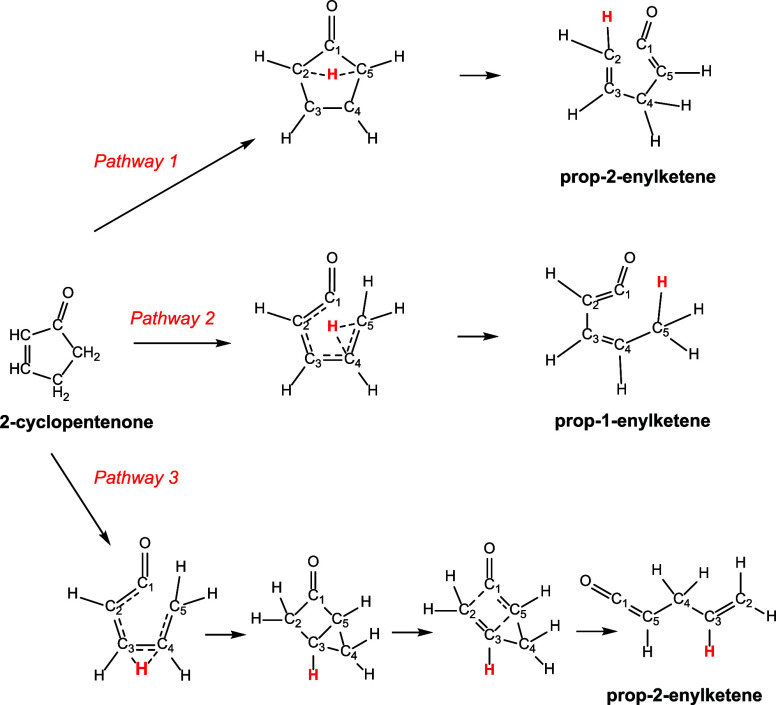
Three possible 2-cyclopentenone decomposition channels, initiated by hydrogen atom migration, leading to propenylketenes. Molecular structures shown in these images are drawn to show the dynamic arrangement of atoms throughout each pathway and not necessarily drawn to represent geometry optimized structures. Geometry optimized structures for all species can be found in the Supporting Information.

**Figure 7:**
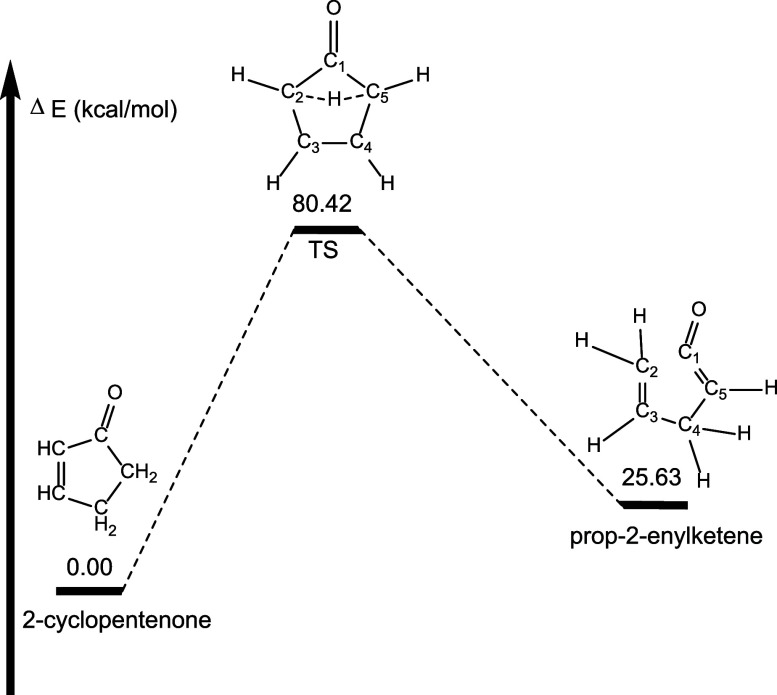
Lowest energy pathway (Pathway 1) for formation of prop-2-enylketene from 2-cyclopentenone. Energetics determined at the B3LYP/6-311++G** level of theory. Note that molecular structures shown in these images are drawn to show the dynamic arrangement of atoms throughout each pathway and not necessarily drawn to represent geometry optimized structures. Geometry optimized structures are shown in Figure 8.

Quantum calculations reveal three unique pathways for hydrogen atom migration in 2-cyclopentenone leading to propenylketenes (Figure 6). Interestingly, the lowest energy pathway, with a barrier of 80.42 kcal mol^–1^, has a hydrogen migrating across the ring from C5 to C2, followed by C2–C1 bond rupture. This produces prop-2-enylketene (or 1,4-pentadien-1-one) that lies 25.63 kcal mol^–1^ higher in energy than 2-cyclopentenone (Figure 7).

**Figure 8:**
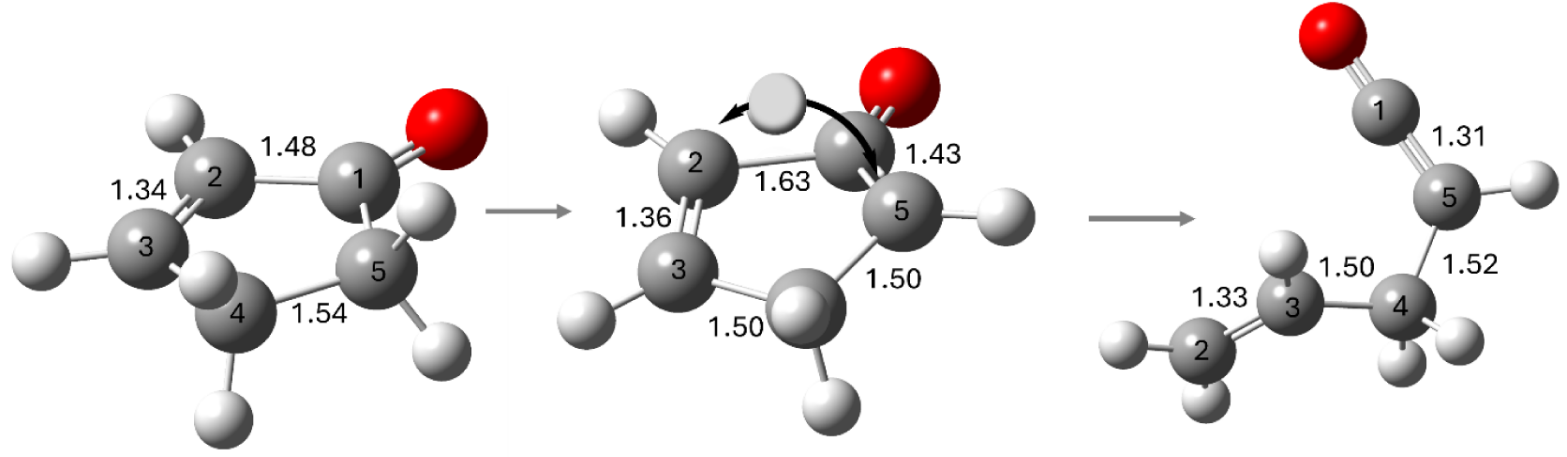
Pathway 1 geometry optimized structures (B3LYP/6-311++G**). This pathway converts 2-cyclopentenone to prop-2-enylketene. For 2-cyclopentenone: ∠O–C1–C2 = 127; ∠O–C1–C5 = 126; ∠C2–C1–C5 = 107; ∠C2–C3–C4 = 113; ∠C3–C4–C5 = 104; ∠1–2–3–4 = 0.011; ∠2–3–4–5 = 0.005; ∠3–4–5–1 = 0.019; ∠4–5–1–2 = 0.025. For prop-2-enylketene ∠5–4–3–2 = 118°.

The geometry optimized structures for Pathway 1 are shown in Figure 8. As expected, 2-cyclopentenone is almost perfectly planar, in spite of having two sp^3^ hybridized carbon atoms in the 5-membered ring (C4 and C5). The hydrogens on those sp^3^ hybridized carbon atoms adopt an eclipsed orientation. In the transition state structure, the H atom moves from C5 to C2, and the 5-membered ring bends slightly out of planarity as the C1–C5 bond shortens to 1.43 Å (from 1.50 Å in 2-cyclopenenone) and the C1–C2 bond lengthens to 1.63 Å. As prop-2-enylketene forms, the C1–C2 bond ruptures and the C5–C4–C3–C2 dihedral angle rotates to 118 degrees.

**Figure 9:**
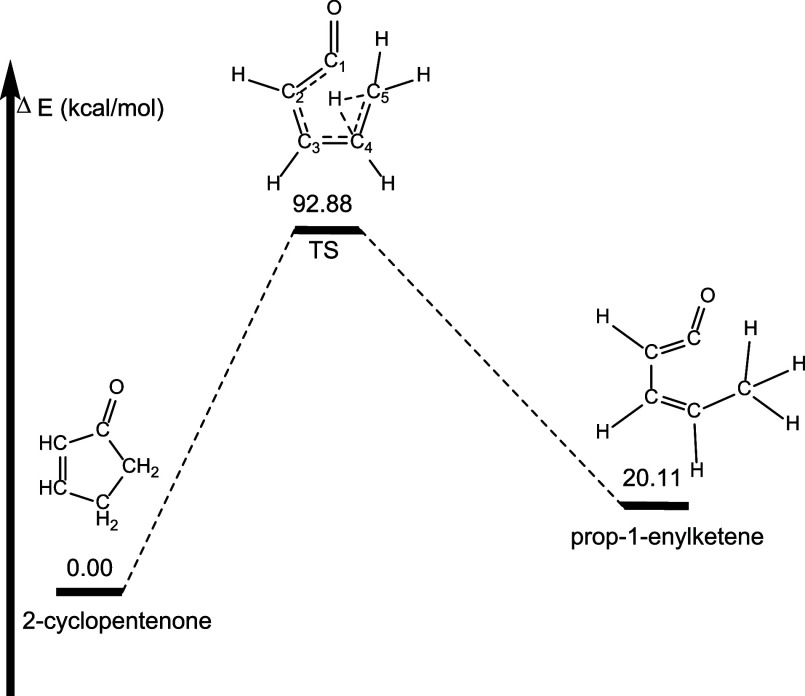
Pathway (Pathway 2) for formation of prop-1-enylketene from 2-cyclopentenone. Energetics determined at the B3LYP/6-311++G** level of theory. Molecular structures shown in these images are drawn to show the dynamic arrangement of atoms throughout each pathway and not necessarily drawn to represent geometry optimized structures. Geometry optimized structures for all species can be found in the Supporting Information.

The second decomposition pathway involves a hydrogen atom migrating from C4 to C5, followed by C1–C5 bond rupture. The barrier for this pathway is 12.46 kcal mol^–1^ higher in energy than for Pathway 1 (92.88 kcal mol^–1^ higher in energy than 2-cyclopentenone) and leads to prop-1-enylketene which lies 5.5 kcal mol^–1^ lower in energy than isomeric prop-2-enylketene found in Pathway 1, and is 20.11 kcal mol^–1^ less stable than 2-cyclopentenone (Figure 9).

**Figure 10:**
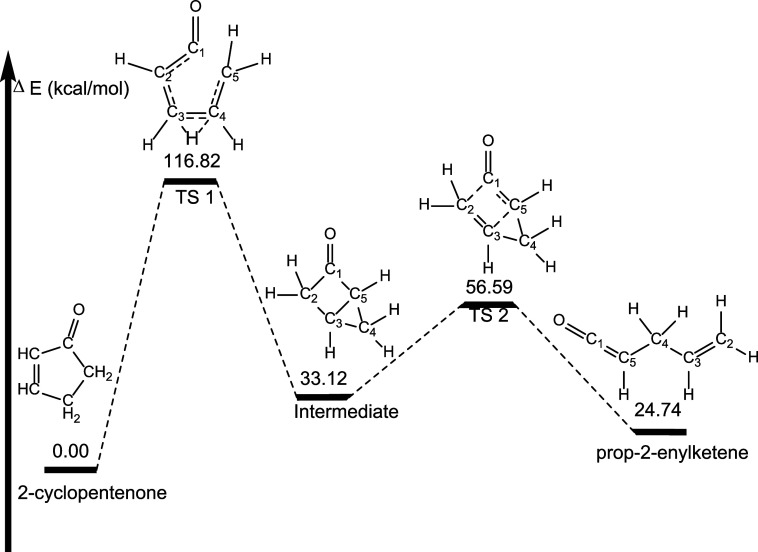
Highest lying pathway (Pathway 3) for formation of prop-2-enylketene from 2-cyclopentenone. Energetics determined at the B3LYP/6-311++G** level of theory. Molecular structures shown in these images are drawn to show the dynamic arrangement of atoms throughout each pathway and not necessarily drawn to represent geometry optimized structures. Geometry optimized structures for all species can be found in the Supporting Information.

The highest energy pathway leading to a propenylketene has a hydrogen atom migrating from C4 to C3 with simultaneous C1 – C5 bond rupture. This process has a barrier of 116.82 kcal mol^–1^ and leads to a bicyclic intermediate that is 33.12 kcal mol^–1^ less stable than 2-cyclopentenenone (Figure 10). This intermediate then undergoes a ring opening via C1–C2 bond rupture with a corresponding barrier of 56.59 kcal mol^–1^ relative to 2-cyclopentenone; 23.47 kcal mol^–1^ relative to the intermediate, leading to a second conformation of prop-2-enylketene. This isomer is 24.74 kcal mol^–1^ less stable than 2-cyclopentenone, and only 0.89 kcal mol^–1^ more stable than the conformer of prop-2-enylketene found via Pathway 1, above.

Our previous work^54^ recorded Ar-matrix FTIR spectra of prop-1-enylketene and prop-2-enylketene, which were each generated via pyrolysis of the appropriate pentenoic anhydride precursor. The antisymmetric C=C=O stretching mode of prop-2-enylketene band^54^ at 2128 cm^–1^ corresponds to the 2129 cm^–1^ band observed here in the spectrum for 1300 K pyrolysis of 2-cyclopentenone. The band at 2123 cm^–1^, also observed following 2-cyclopentenone pyrolysis, could be reasonably assigned to either prop-1-enyl-ketene or prop-2-enylketene.^54^

**Figure 11:**
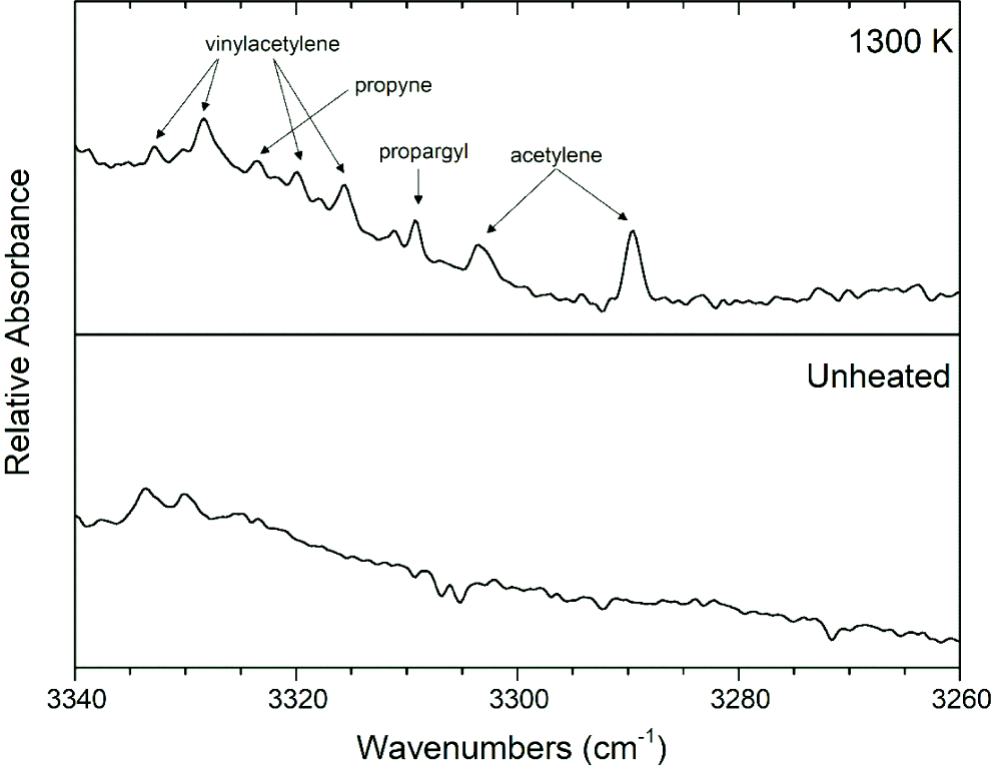
Matrix-isolation FTIR spectrum collected following 1300 K pyrolysis of 0.3% 2-cyclopentenone in argon (upper trace) compared to that of unheated 0.3% 2-cyclopentenone in argon.

**Figure 12:**
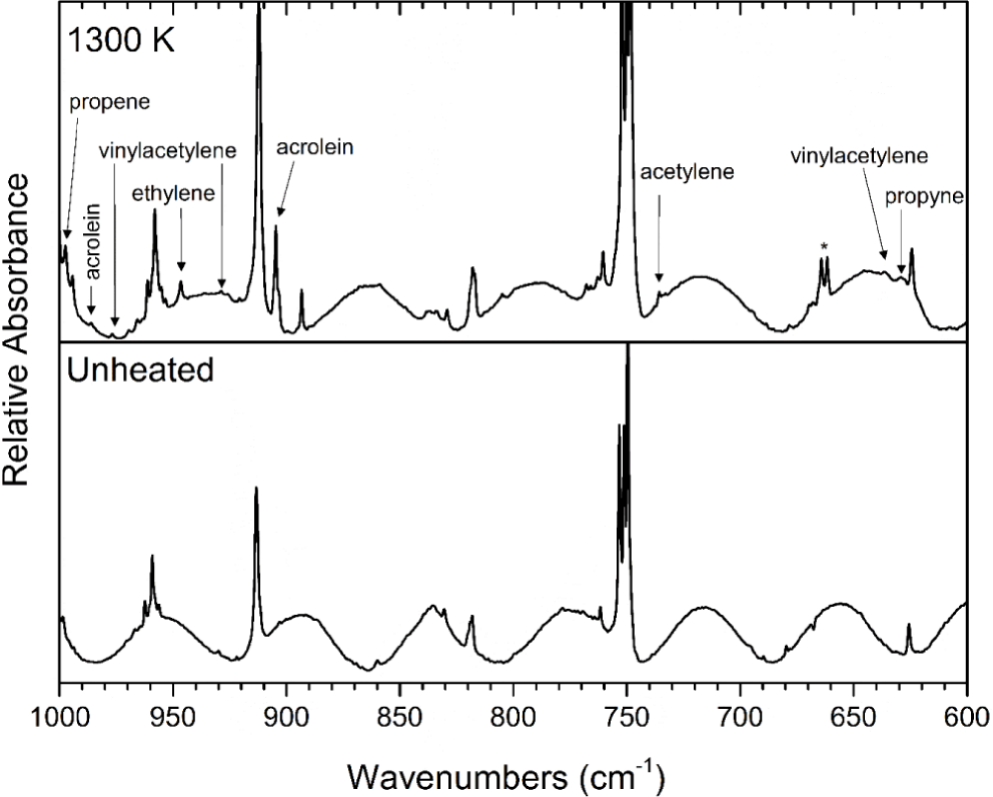
Matrix-isolation FTIR spectrum collected following 1300 K pyrolysis of 0.3% 2-cyclopentenone in argon (middle trace) compared to that of unheated 0.3% 2-cyclopentenone in argon (upper trace). The top trace shows the difference spectrum, which clearly shows the assigned products. The asterisk indicates background CO_2_.

Acetylene bands were observed at 3302, 3288, and 737 cm^–1^ (Figures 11 and 12), in accordance with the literature.^51^ Another proposed mechanism of 2-cyclopentenone dissociation, assuming a unimolecular pathway, is the molecule thermally decomposing to form acetylene, carbon monoxide (CO), and ethylene (H_2_C=CH_2_). This pathway would potentially involve a simple ring opening mechanism with C–C bond cleavage between C1 and C2, C3 and C4, and C5 and C1. Ethylene is shown by the observed bands, both monomer and dimer, at 3114, 3105, 3083, 2995, 2979, 1440, and 947 cm^–1^ (Figures 4, 5, and 12), which match band values from the previous literature.^55−57^

**Figure 13:**
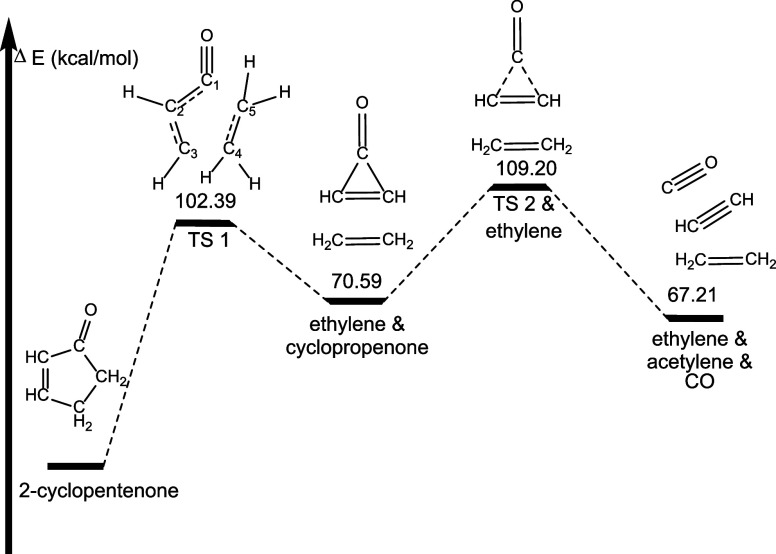
Pathway for formation of ethylene, acetylene and CO from 2-cyclopentenone decomposition. Energetics determined at the B3LYP/6-311++G** level of theory. Molecular structures shown in these images are drawn to show the dynamic arrangement of atoms throughout each pathway and not necessarily drawn to represent geometry optimized structures. Geometry optimized structures for all species can be found in the Supporting Information.

Computationally, we see 2-cyclopentenone decomposing to ethylene, acetylene, and CO via a two-step mechanism. This pathway is initiated via a simultaneous C1–C5 and C3–C4 bond rupture corresponding to a reaction barrier of 102.39 kcal mol^–1^. This leads to a minimum on the potential energy surface containing cyclopropenone and ethylene, which lies 70.59 kcal mol^–1^ higher in energy than 2-cyclopropenone (Figure 13). This is separated from ethylene, acetylene, and CO by a transition state corresponding to the simultaneous rupture of C1–C2 and C1–C3, releasing CO and forming acetylene.

**Figure 14:**
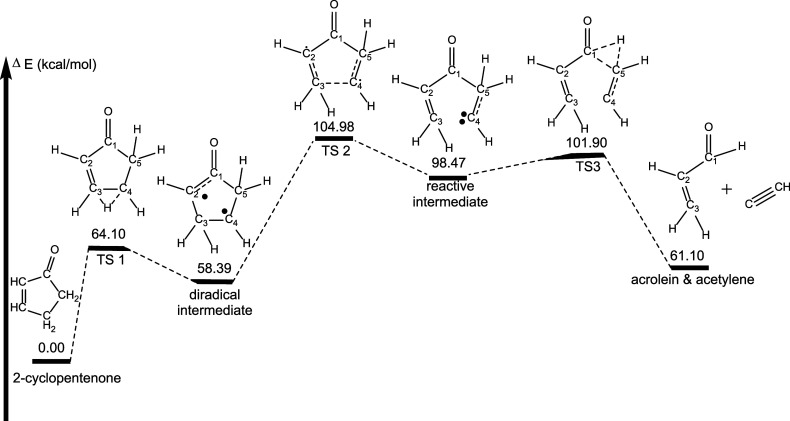
Pathway for formation of acrolein and acetylene from cyclopentenone decomposition. Energetics determined at the B3LYP/6-311++G** level of theory. Molecular structures shown in these images are drawn to show the dynamic arrangement of atoms throughout each pathway and not necessarily drawn to represent geometry optimized structures. Geometry optimized structures for all species can be found in the Supporting Information.

Acrolein (H_2_C=CHCHO) vibrational bands were observed at 1156, 986, and 905 cm^–1^ (Figures 5 and 12), which match bands reported in the literature.^58,59^ Unfortunately, the strongest bands of acrolein, 1708 cm^–1^ for the *cis* isomer and 1715 cm^–1^ for the *trans*, overlap the carbonyl stretch of unreacted 2-cyclopentenone and could not be distinguished here. If 2-cyclopentenone is decomposing unimolecularly, then one proposed pathway is the molecule thermally decomposing to form acrolein and acetylene (HC=CH). This mechanism would be feasible if two hydrogen atom migrations occurred, from C5 to C1 and from C4 to C3, coupled with ring opening, thus forming the two products. We were unable to find a concerted pathway for this decomposition; however, we were able to identify a unimolecular multistep pathway that results in these products (Figure 14). The overall barrier for this fifth pathway is ∼105 kcal/mol, and is initiated in a similar fashion to pathway 3, i.e., by a hydrogen migration from C4 to C3 but without simultaneous C1–C5 bond rupture. Instead, a diradical intermediate is formed that is ∼60 kcal/mol less stable than cyclopentenone, and the barrier for this first step is only 64 kcal/mol making this first step a competitive reaction channel. The resulting diradical must surmount a barrier of 105 kcal/mol (relative to cyclopentenone) in order to undergo C3–C4 bond rupture, forming an acyclic intermediate with a carbene-like C4 atom, which lies ∼98 kcal/mol higher in energy than cyclopenenone. The barrier separating this intermediate from acrolein and acetylene is only ∼3 kcal/mol.

Vinylacetylene (CH_2_=CHC≡CH) was detected at 3332, 3327, 3319, 3315, 3122, 977, 929, and 636 cm^–1^ (Figures 4, 11 and 12), which match the values from the literature.^60^ For the formation of vinylacetylene, two potential unimolecular pathways could occur. The first pathway leads to the formation of vinylacetylene, molecular hydrogen (H_2_), and carbon monoxide (CO). Because H_2_ does not have a dipole moment, it is not infrared active and would not be visible in the FTIR spectra. The second pathway would lead to the formation of formaldehyde (H_2_CO); however, there were no vibrational bands consistent with formaldehyde^61^ in the spectra.

Propene (H_2_C=CHCH_3_) was evidenced by the observed bands at 3086, 3038, 2986, 1440, 1044, and 998 cm^–1^ (Figures 4, 5 and 12), which match bands from the literature.^55^ Propyne was evidenced^62^ at 3323 and 629 cm^–1^ in Figures 11 and 12. Propargyl radical (•CH_2_C≡CH) was found at 3309 cm^–1^ (Figure 11) While many radicals can be difficult to observe, the propargyl radical is resonance stabilized and has been observed with matrix-isolation FTIR detection in similar pyrolysis experiments on other molecules.^63,64^ Another band would be expected^65^ at 686 cm^–1^, but unreacted 2-cyclopentenone appears there in the spectrum. (Figure 12) For the formation of propargyl radicals, one potential unimolecular pathway involves C–C bond cleavage of 2-cyclopentenone between C1 and C2, and also between C4 and C5. In addition, a loss or migration of a hydrogen atom from C3 would also need to occur to make propargyl. Along with propargyl radicals, this mechanism would also form a coproduct with the molecular formula C_2_H_3_O, however, a radical such as acetyl or vinoxy is unlikely to be sufficiently stable for detection in these experiments. The propargyl radicals are of particular significance since they indicate that 2-cyclopentenone might be a precursor to polycyclic aromatic hydrocarbons (PAH), which lead to the formation of soot in certain high-temperature environments;^66,67^ this directly affects the environmental impact of the production and use of certain biofuels.

Finally, the isomerization of 2-cyclopentenone should be considered as a possibility, particularly to 3-cyclopentenone. While we were able to reproduce the isomerization barrier height computed previously^35^ (∼70 kcal/mol; see Figure S2), it was not observed in our experiments. Many of the FTIR bands for 2-cyclopentenone and 3-cyclopentenone overlap; however, there are four bands that appear unique to 3-cyclopentenone: 1739, 1266, 1185, and 1181 cm^–1^. These bands were not observed in the spectra collected following pyrolysis of 2-cyclopentenone so it is concluded that isomerization is negligible. (Figure S1)

## Conclusions

The pyrolysis of 2-cyclopentenone has been studied experimentally in the gas phase and computationally. Carbon monoxide, ketene, prop-2-enylketene and/or prop-1-enylketene, vinylacetylene, ethylene, propene, acrolein, acetylene, propyne, and propargyl radical were all identified via matrix-isolation FTIR. Many of these products can be linked to unimolecular reactions, although the possibility of bimolecular reactions must also be considered. Computationally, we were able to identify five different unimolecular decomposition channels. Three of the four channels involve a hydrogen atom migration in the cyclopentenone ring followed by bond rupture, producing propenylketene, while the fourth and fifth pathways corresponds to a multistep decompositions where either two simultaneous C–C bonds rupture leading to carbon monoxide, acetylene and ethylene (pathway 4), or hydrogen migration generates a diradical intermediate that rearranges to a carbene-like species that ruptures into acrolein and acetylene (pathway 5).

## Supporting Information Available

Full Gaussian software citation, image comparing 2-cyclopentenone with 3-cyclopentenone FTIR spectra, potential energy surface for the isomerization of 2-cyclopentenone to 3-cyclopentenone, and Cartesian coordinates for all species (PDF)
