# Supplementary material for: Thermal Decomposition of 2-Cyclopentenone
Source: J Phys Chem A. 2024 Oct 15;128(42):9226–34. doi: 10.1021/acs.jpca.4c05532 (PMC11514027; doi:10.1021/acs.jpca.4c05532)
Supplement: Supplementary file 1 — jp4c05532_si_001.pdf [file jp4c05532_si_001.pdf]

Thermal Decomposition of 2-cyclopentenone

Kathryn Narkin[a], Heather R. Legg[a], Glenna J. Brown[a], Khaled El-Shazly[a], Thaddeus D. Martin[a], Mia Jarrell[a], Laura R. McCunn[a]*, Zhijian Chen[b], Carol A. Parish[b†]

[a]Department of Chemistry, Marshall University, 1 John Marshall Dr. Huntington, West Virginia 25755

[b]Department of Chemistry, University of Richmond, Gottwald Center for the Sciences, Richmond, VA 23173

*corresponding author (experimental): mccunn@marshall.edu, 304-696-2319

[†]corresponding author (computational): cparish@richmond.edu, 804-484-1548

Full Gaussian 16 citation: Gaussian 16, Revision C.01, Frisch, M. J.; Trucks, G. W.; Schlegel, H. B.; Scuseria, G. E.; Robb, M. A.; Cheeseman, J. R.; Scalmani, G.; Barone, V.; Petersson, G. A.; Nakatsuji, H.; Li, X.; Caricato, M.; Marenich, A. V.; Bloino, J.; Janesko, B. G.; Gomperts, R.; Mennucci, B.; Hratchian, H. P.; Ortiz, J. V.; Izmaylov, A. F.; Sonnenberg, J. L.; Williams-Young, D.; Ding, F.; Lipparini, F.; Egidi, F.; Goings, J.; Peng, B.; Petrone, A.; Henderson, T.; Ranasinghe, D.; Zakrzewski, V. G.; Gao, J.; Rega, N.; Zheng, G.; Liang, W.; Hada, M.; Ehara, M.; Toyota, K.; Fukuda, R.; Hasegawa, J.; Ishida, M.; Nakajima, T.; Honda, Y.; Kitao, O.; Nakai, H.; Vreven, T.; Throssell, K.; Montgomery, J. A., Jr.; Peralta, J. E.; Ogliaro, F.; Bearpark, M. J.; Heyd, J. J.; Brothers, E. N.; Kudin, K. N.; Staroverov, V. N.; Keith, T. A.; Kobayashi, R.; Normand, J.; Raghavachari, K.; Rendell, A. P.; Burant, J. C.; Iyengar, S. S.; Tomasi, J.; Cossi, M.; Millam, J. M.; Klene, M.; Adamo, C.; Cammi, R.; Ochterski, J. W.; Martin, R. L.; Morokuma, K.; Farkas, O.; Foresman, J. B.; Fox, D. J. Gaussian, Inc., Wallingford CT, 2016.

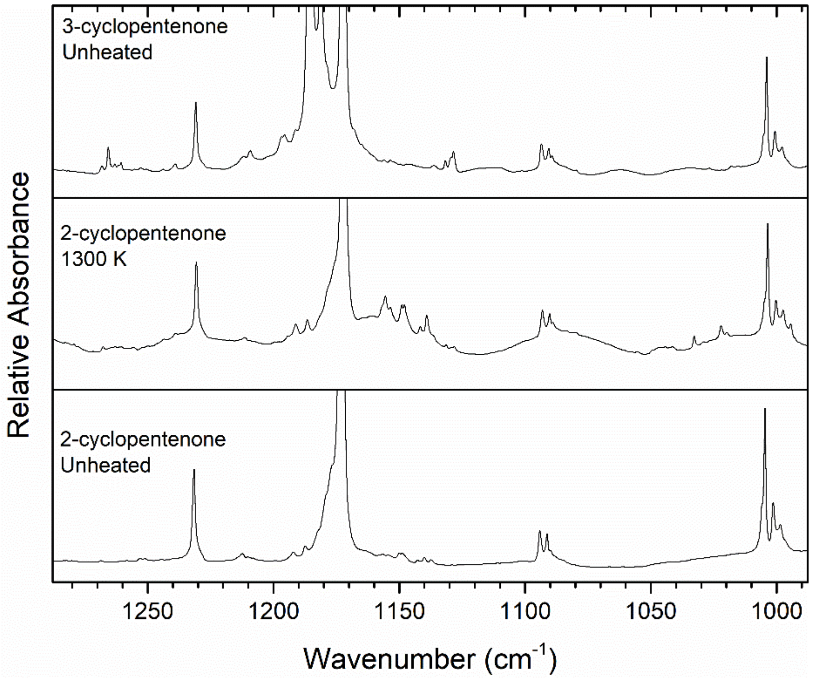

Figure S1. Argon-matrix FTIR spectra of 2-cyclopentenone (bottom), 3-cyclopentenone (top) confirming negligible isomerization of 2-cyclopentenone to 3-cyclopentenone during pyrolysis (middle).

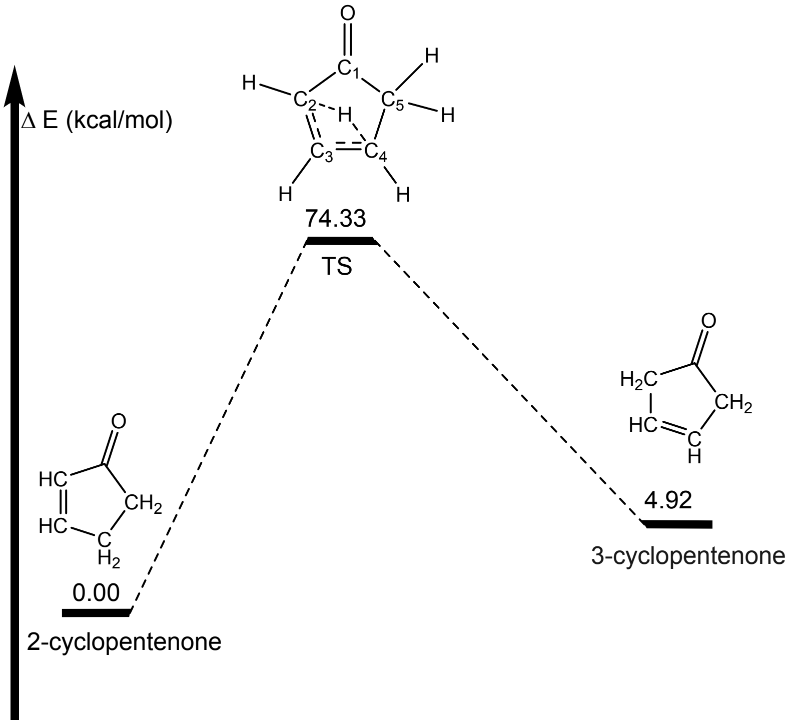

Figure S2. Pathway for isomerization of 2-cyclopentenone to 3- cyclopentenone. Energetics determined at the B3LYP/6-311++G** level of theory. Molecular structures shown in these images are drawn to show the dynamic arrangement of atoms throughout each pathway and not

necessarily drawn to represent geometry optimized structures. Geometry optimized structures for all species can be found in the Supporting Information.

Sample Input File for Reactant and Product Optimization:

```
%chk      = filename.chk
%mem      = 50GB
%nproc    = 10
# opt freq b3lyp/6-311++g(d,p)

Title Card that explains this job

0 1 #spin and multiplicity

#XYZ coordinates inserted here
```

Sample Input File for Transition State Structure:

```
%chk      = filename.chk
%mem      = 50GB
%nproc    = 10
# opt=(calcfc,ts,noeigen,MaxCyc=200,MaxStep=200) freq b3lyp/6-311++g(d,p)

Title Card that explains this job

0 1 #spin and multiplicity

#XYZ coordinates inserted here
```

XYZ Coordinates for Reactants, Transition States, Intermediates, Products:

2-cyclopentenone:

C	-1.3577640	0.8188950	-0.0632590
H	-2.2231530	1.4214560	-0.2447050
H	-0.0854240	-1.3203010	-1.3470180
C	-1.3635030	-0.6872950	0.2825990
H	-1.3790110	-0.7792270	1.3485300
C	-0.0235400	-1.1736690	-0.2889210
H	0.3004490	-2.0896180	0.1593720
C	-0.0611760	1.2581450	-0.0722250
H	0.2517820	2.2808250	-0.1051260
C	0.8721650	0.0323800	-0.0265600
O	2.1159410	0.0291390	0.1647030
H	-2.1892590	-1.2369860	-0.1184830

TS (2 to 3 cyclopentenone):

C	-1.4018520	0.7133220	0.0061490
H	-2.2537930	1.3845970	0.0038440
H	0.1712250	-1.8553100	-0.8309200
C	-1.4796260	-0.6088500	0.0433140
H	-0.5143130	0.1957130	0.8013700
C	-0.0446850	-1.2235130	0.0338170
H	0.2034560	-1.8058420	0.9243130
C	0.0347000	1.2282650	-0.0345880
H	0.4104260	2.2422100	-0.0691120
C	0.8544780	0.0204850	-0.0172790
O	2.0662020	0.0201420	-0.0404810
H	-2.3247110	-1.1007620	-0.6941230

3-cyclopentenone:

C	-1.4345830	0.6689590	-0.0001250
H	-2.3265900	1.2882780	0.0000380
H	0.1785010	-1.8704040	-0.8780360
C	-1.4345060	-0.6691780	-0.0001300
H	-2.3265140	-1.2884720	-0.0003810
C	-0.0439690	-1.2477150	0.0001820
H	0.1782410	-1.8701820	0.8786350
C	-0.0441220	1.2478280	0.0001800
H	0.1779930	1.8701660	-0.8783950
H	0.1778850	1.8706120	0.8783910
C	0.8577310	0.0000670	0.0001160
O	2.0671480	0.0000290	-0.0001980

Path 1:

TS:

C	0.9250160	-0.0794020	-0.0398590
O	2.1238890	0.0329100	-0.1884410
C	-0.0921050	1.1825540	0.1608570
H	0.2881000	2.1943150	0.2523500
C	-1.3664570	0.7863640	-0.1185230
H	-2.2381380	1.4112090	-0.2748600
C	-1.3936800	-0.7128860	-0.1192140
H	-2.1775360	-1.1202880	0.5325890
H	-1.6466370	-1.0481550	-1.1409610
C	-0.0007530	-1.1418160	0.2387760
H	0.2924040	-2.1871060	0.2358680
H	0.0585710	0.2778630	1.1703250

Product (Prop-2-Enylketene):

C	1.7411030	-0.0440370	-0.0204460
O	2.5174640	-0.8947000	0.1552670
C	-2.6778600	-0.5723180	0.0517570
H	-3.1507360	-1.4856040	-0.2873290
C	-1.3961990	-0.3332370	-0.2115060
H	-0.8330630	-1.0641930	-0.7887800
C	-0.5932620	0.8706920	0.2066690
H	-1.0718710	1.7598120	-0.2141210
H	-0.6797770	0.9955240	1.2968080
C	0.8653590	0.9155210	-0.2186530
H	1.2482720	1.8047550	-0.7071820
H	-3.2873900	0.1275780	0.6115390

Path 2:

TS:

C	1.0426760	0.1005670	-0.0584440
O	2.1876580	-0.2229560	-0.0609130
C	0.1679610	1.1854830	0.0965480
H	0.5791370	2.1813770	-0.0405430
C	-1.2140140	0.9304630	0.0297460
H	-1.9268530	1.7487670	0.0112020
C	-1.6215870	-0.4039540	-0.0656860
H	-2.6453330	-0.7445320	-0.1642090
H	-0.7202100	-0.7309870	-0.9548930
C	-0.4308950	-1.3245260	0.1201570
H	-0.4909230	-2.3599530	-0.2102690
H	0.0380780	-1.2392240	1.1120960

Product (Prop-1-Enylketene):

C	1.0290540	0.1045440	-0.0521040
O	2.1733240	-0.2020810	-0.0670770
C	0.1505360	1.1843430	0.0977220
H	0.5548970	2.1822690	-0.0224890
C	-1.2268700	0.9144440	0.0246350
H	-1.9503650	1.7199320	0.0014100
C	-1.6116980	-0.4230200	-0.0685790
H	-2.6242640	-0.7859450	-0.1660570
H	-0.6852660	-0.7291990	-0.9465470
C	-0.3937770	-1.3150540	0.1242100
H	-0.4144840	-2.3482790	-0.2089450
H	0.0494140	-1.2136660	1.1239390

Path 3:

TS1 with H migration:

C	0.8315100	-0.1243760	0.0303220
O	2.0300600	-0.1655050	-0.0717110
C	-0.1755360	-1.2058670	-0.0475960
H	0.1138590	-2.2505170	-0.1129940
C	-1.4189090	-0.6120350	0.2435650
H	-1.9753210	-0.6645170	1.1738070
C	-1.1348440	0.6664510	-0.4386590
H	-2.3925230	0.0198600	-0.4834360
H	-1.0017680	0.5754780	-1.5131800
C	0.0026430	1.2886300	0.2733450
H	-0.1422160	1.4488580	1.3415840
H	0.5282990	2.1180610	-0.1979550

Intermediate:

C	0.8884500	-0.1161480	0.0314350
O	2.0461960	-0.4288460	0.0978740
C	-0.3457550	-0.8341200	-0.4104850
H	-0.3950030	-1.6698850	-1.0968650
C	-1.4974560	-0.5154630	0.5406650
H	-1.3040640	-0.3942840	1.6023670
C	-1.0860950	0.5378720	-0.4189700
H	-2.4074980	-1.0589830	0.3132570
H	-1.7269360	0.9035440	-1.2092960
C	0.1668150	1.2554290	0.1101130
H	0.0839000	1.7346100	1.0889110
H	0.6242740	1.9503400	-0.5979150

TS2 with ring opening:

C	-0.8568250	-0.2571500	-0.0708450
O	-2.0454870	-0.2140330	0.0886740
C	0.1947620	-1.1622770	-0.0223710
H	0.0430030	-2.2213450	0.1601830
C	1.5481910	-0.5002640	-0.1901190
H	1.8853960	-0.3386560	-1.2197940
C	1.0090990	0.7110780	0.4796180
H	2.3242660	-1.0207370	0.3728810
H	1.1281300	0.8458120	1.5479730
C	0.0106800	1.3541480	-0.2670670
H	0.1568760	1.4598720	-1.3391920
H	-0.6092190	2.1141150	0.1932610

Product (prop-2-enylketene):

O	-2.8150960	-0.6488270	0.1955500
C	-1.8001850	0.0314770	-0.1056330
C	-0.7072030	0.7641130	-0.4299840
H	-0.7325160	1.4160300	-1.2780770
C	0.5712520	0.6583790	0.4220560
H	1.0975800	1.5895110	0.3925180
H	0.3078330	0.4295060	1.4335540
C	1.4713160	-0.4580830	-0.1391910
H	1.0337930	-1.3007910	-0.6324620
C	2.8175130	-0.3732430	-0.0083420
H	3.4428820	-1.1489660	-0.3983000
H	3.2550370	0.4694660	0.4849280

Path 4:

TS 1 forming ethylene and cyclopentenone:

C	1.0073960	-0.5935760	0.0000660
O	1.1116820	-1.7360470	-0.0002340
C	1.3658920	0.7430440	0.0001780
H	2.4647780	0.7380990	0.0003010
C	0.5640240	1.8619320	0.0000230
H	1.2244220	2.7448680	-0.0001490
C	-1.9840710	0.5571340	-0.0003020
H	-2.0821770	1.1244490	0.9158830
H	-2.0816340	1.1236480	-0.9170400
C	-1.7821000	-0.7648960	0.0003210
H	-1.7225850	-1.3326470	-0.9224160
H	-1.7231000	-1.3318640	0.9235870

Ethylene:

C	0.9583525	-0.0682062	0.0601115
H	0.4233525	0.5454514	0.7544452
H	0.4233525	-0.6818639	-0.6342222
C	2.3187525	-0.0682062	0.0601115
H	2.8537525	0.5454514	0.7544452
H	2.8537525	-0.6818639	-0.6342222

Cyclopentenone:

C	-0.5915510	0.1397560	-0.0003270
O	-1.6547500	-0.2223260	0.0003710
C	0.7095780	0.5417020	-0.0000890
H	0.9176050	1.5992600	0.0001130
C	1.5088770	-0.6004460	-0.0001460
H	2.5589700	-0.3067220	0.0002940

TS 2 forming acetylene and CO:

C	0.7203680	0.3813790	-0.0000110
O	1.6212190	-0.3260890	0.0000050
C	-0.9228310	0.5765270	0.0000050
H	-1.1557370	1.6295700	0.0000110
C	-1.3827740	-0.6021070	-0.0000050
H	-2.3025920	-1.1556520	0.0000120

Acetylene:

C	0.9052445	0.0127958	0.0292859
H	1.9752445	0.0127958	0.0292859
C	-0.3986115	0.0127958	0.0292859
H	-1.4686115	0.0127958	0.0292859

CO:

C	1.0905113	-0.0345032	0.0248441
O	2.3614113	-0.0345032	0.0248441

Path 5:

TS 1 moving H from C4 to C3:

C	-0.8731090	0.0663720	0.1001810
C	0.1236000	1.1884520	0.2295430
H	-0.0837030	2.1966920	0.5598330
C	1.3379580	0.6453740	0.0323570
H	2.0555620	-0.6281780	-1.1699850
H	2.2608450	1.2170890	0.0225830
O	-1.9722070	0.0882580	-0.3891480
C	-0.0142980	-1.1169290	0.5216000
H	0.3388940	-0.8307460	1.5156350
C	1.2480730	-0.7751750	-0.4107930
H	0.6175080	-0.5634600	-1.3112340
H	-0.3447950	-2.1460230	0.6590150

Diradical ring structure (radicals on C4 and C2):

C	-0.8769420	-0.0659380	-0.0152480
C	0.0519420	-1.1876680	-0.0519390
H	-0.2553000	-2.2239330	-0.0766880
C	1.2986990	-0.7110610	0.0273780
H	2.5589520	0.6212630	0.0456570
H	1.8536370	-1.1134400	-0.7942280
O	-2.0885120	-0.0702750	0.0450480
C	-0.0507620	1.2204430	-0.0320960
H	-0.3463530	1.7739810	-0.9304620
C	1.4909480	0.8664690	0.0138330
H	-0.3186840	1.8547210	0.8121160
H	1.7325330	-1.0838640	0.9316490

TS breaking C3 - C4 bond:

C	0.8121990	0.0968960	0.0205470
C	0.0482770	-1.2362990	-0.0824740
H	0.3383950	-1.8768500	0.7240160
C	-1.4661120	-0.9674810	-0.0052550
H	-1.9193360	-0.1708140	-0.1086050
H	-1.9969020	-1.8937560	-0.0772090
O	2.2185040	-0.1529710	-0.0485560
C	-0.0104640	1.3150770	0.0418950
H	0.1384730	1.8566740	0.9526030
C	-1.4451440	0.7605220	-0.0340100
H	-2.1440320	1.5652560	0.0601090
H	0.2028340	1.9309690	-0.8066810

Anionic structure (anion on C4):

C	0.8287250	-0.0676930	0.0211810
C	-0.0659390	-1.2420660	-0.0840120
H	0.3742710	-2.1883300	-0.3749820
C	-1.3889720	-1.3610500	0.1178510
H	-1.8069570	-0.4747330	0.6312430
H	-2.1365060	-2.1157070	-0.0944040
O	2.0350190	-0.1315920	-0.0535590
C	0.0500360	1.2505630	0.2137470
H	0.1382000	1.5170030	1.2779230
C	-1.3238580	1.4908050	-0.4293080
H	-2.0505460	1.8845950	0.3118840
H	0.6014310	2.0065550	-0.3599380

TS breaking C1 - C5:

C	1.0967600	0.2596620	0.0476180
O	2.3367030	0.2257400	-0.1644250
C	0.3744490	-0.9551360	0.2582800
H	0.6068550	-1.4531300	1.1984640
C	-0.8732080	-1.1277190	-0.3100340
H	-1.4972230	-1.9355480	0.0578150
C	-1.7740680	0.2847990	0.0631610
H	-2.7449110	-0.1630030	0.1088690
C	-1.0567010	1.2971390	0.0782170
H	-0.8858960	2.3533770	0.1244930
H	-1.3591740	-0.3582490	-1.0678640
H	0.5833290	1.1981610	0.0701690

Acrolein:

C	0.6565450	0.3904320	-0.0420450
C	-0.5556560	-0.4566590	0.0183280
H	-0.4236090	-1.5313710	-0.0193820
C	-1.7557070	0.1296750	-0.0070720
H	-1.8914620	1.1941530	0.2618100
H	-2.6946450	-0.3972550	-0.1258480
O	1.7969230	-0.1372110	0.0265180
H	0.5632380	1.4514700	-0.1439880
